# Case Report: Diagnosis of *Nocardia asteroides* infection using metagenomic next-generation sequencing of lymph node puncture tissue

**DOI:** 10.3389/fmed.2025.1626685

**Published:** 2025-09-12

**Authors:** Ya Min Yang, Jing Guo, Ning Ning Dang

**Affiliations:** Department of Dermatology, Shandong Provincial Hospital Affiliated to Shandong First Medical University, Jinan, China

**Keywords:** *Nocardia asteroides*, nocardiosis, muscle abscess, metagenomic next-generation sequencing, LRBA gene mutation

## Abstract

The Nocardia genus is an aerobic, Gram positive, opportunistic pathogen that primarily affects cell-mediated immunosuppressed patients. The clinical manifestation of nocardiosis varies widely, making it challenging to diagnose. In this report, we describe a 48 year old woman with a muscular abscess caused by *Nocardia asteroides*. Venous blood, skin biopsy specimen, muscle tissue, and inguinal lymph node puncture tissue cultures yielded negative results. Using metagenomic next-generation sequencing (mNGS), the pathogen was identified as *Nocardia asteroides*. Whole-exome sequencing of the peripheral blood showed that the patient had a monoallelic mutation in the lipopolysaccharide-responsive beige-like anchor protein (LRBA) gene. The mNGS detected *Nocardia asteroides* in the patient, and the administration of accurate treatment led to her complete recovery.

## Introduction

The genus *Nocardia* is a ubiquitous group of environmental bacteria that usually cause opportunistic infections in immunocompromised hosts. *Nocardia* is widely distributed in plants, gardens, and soil and is classified into more than 80 species, over 50 of which can cause human diseases (1). Nocardiosis is an infectious disease caused by *Nocardia* species, and patients with compromised cell-mediated immunity are at a higher risk of developing this condition (1, 2). Nocardiosis exhibits variable clinical manifestations, complicating its diagnosis. Traditional diagnostic methods rely on culture techniques, which have limitations, such as low sensitivity (3). Recently, metagenomic next-generation sequencing (mNGS) has emerged as a superior alternative, offering high sensitivity and specificity for diagnosis. This advanced technique enables the detection of rare and hard-to-identify pathogens directly from clinical samples, demonstrating significant advantages in diagnosing infectious diseases such as nocardiosis (4). Here, we report a case of intramuscular abscesses caused by *Nocardia asteroides*, which was accurately identified using mNGS. Owing to the rapidity and high sensitivity of this diagnostic approach, the patient received timely antimicrobial therapy and full recovered. We present this case to highlight how a *Nocardia* infection can progress from superficial skin and soft tissue involvement to deeper muscle invasion, emphasizing the imperative for timely diagnosis and therapeutic intervention.

## Case presentation

A 48-year-old woman presented to our clinic with a history of recurrent swelling, painful erythema on the right inner thigh, and fever lasting more than 1 year. Twelve months ago, the patient developed a low-grade fever that did not subside under despite unsupervised self-medication with amoxicillin and cefuroxime. Subsequently, an erythematous lesion with tenderness appeared on the medial aspect of the right thigh. Despite treatment with antibiotics (non-specified) and corticosteroids, the patient developed an overlying ecchymosis accompanied by significant local pain, followed shortly by episodes of fever (38.5 °C). Sporadic intake of antibiotics and prednisone provided some symptomatic relief, despite the persistence of the cutaneous lesion. Over the last 2 months, the patient noticed a progressive worsening of the local pain. She had no known underlying immunosuppressive disease.

**Figure 1:**
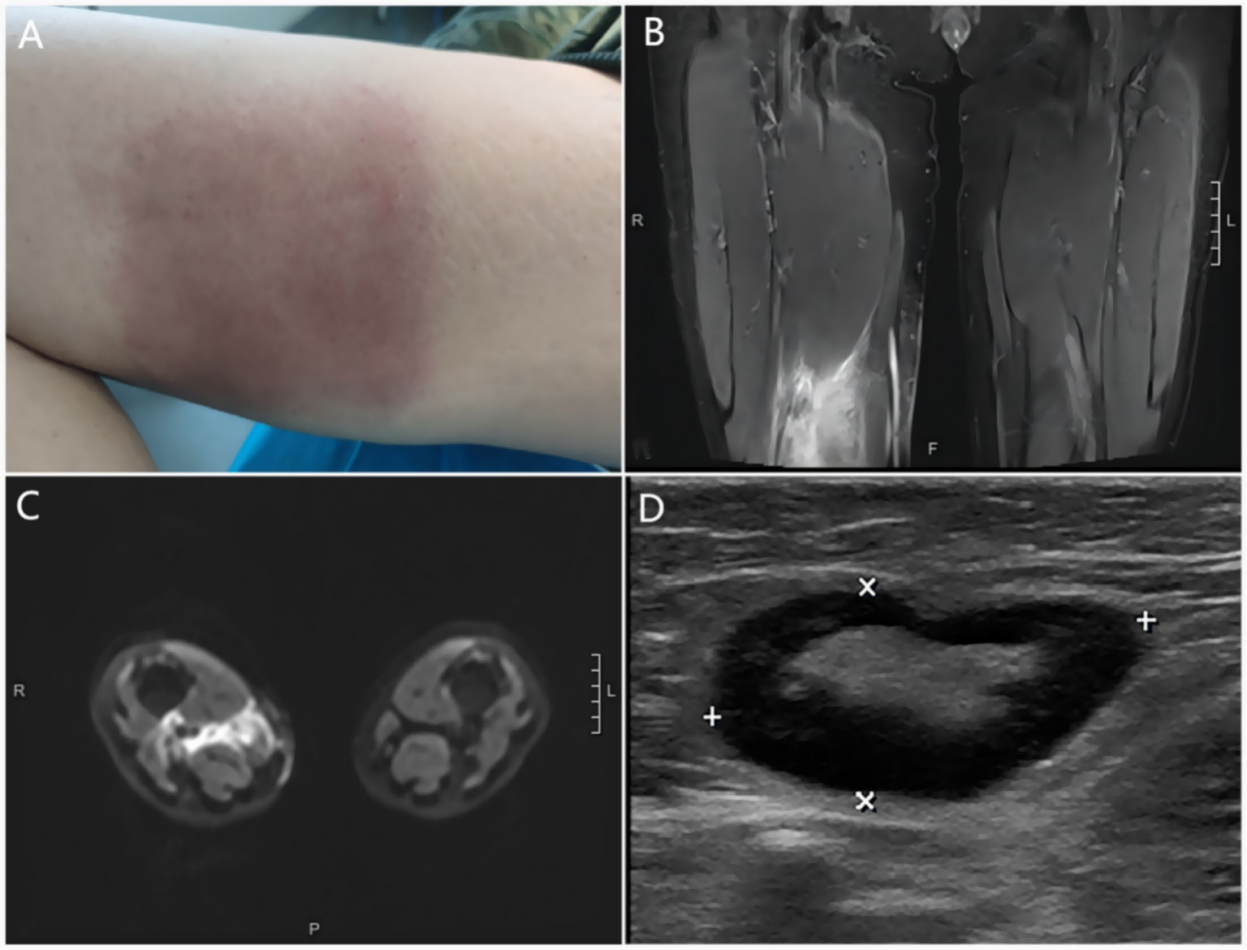
**(A)** 15 × 15 cm square-shaped edematous, infiltrative, well-demarcated erythema on the right medial femur with a tough texture. **(B)** Coronal MRI of the lower limbs showing abnormal signals in the right lower posterior femoral muscle group and muscle spaces. **(C)** Transverse axial MRI of the lower limbs showing abnormal signals in the right lower posterior femoral muscle group and muscle spaces. **(D)** Ultrasonography of the right groin showing an enlarged lymph node with a clear border (2.7 × 1.3 cm).

**Figure 2:**
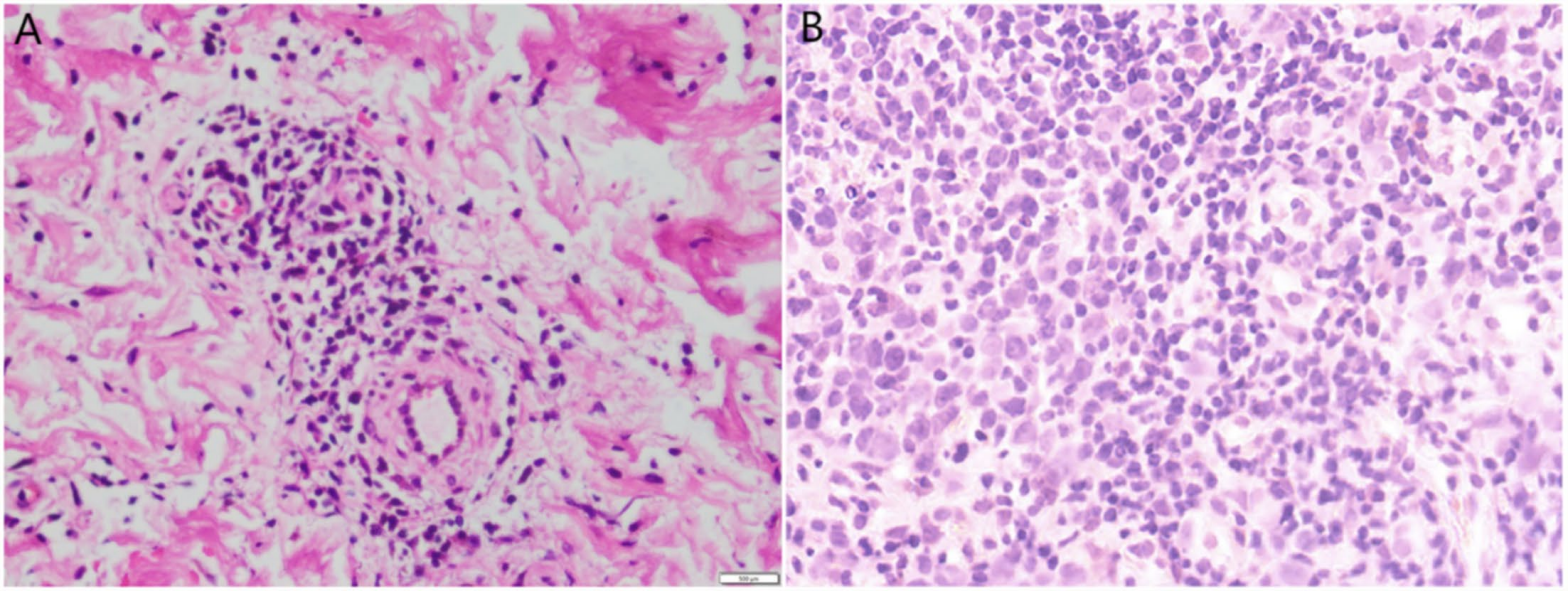
**(A)** Skin biopsy revealed lymphocytic infiltration around small blood vessels in the dermis (H & E, 400×). **(B)** Lymph node aspiration tissue biopsy revealed proliferative lesions of lymphoid tissue (H & E, 400×).

Upon examination, the patient’s temperature was 39.3 °C, her pulse was 110 beats per min, blood pressure measured 129/76 mm Hg, and her respiratory rate was 21 breaths per min. Physical examination revealed enlarged cervical and inguinal lymph nodes. The dermatological examination revealed a 15 × 15 cm square-shaped edematous, infiltrative, well-demarcated erythema on the medial aspect of the right thigh, with a tough texture (Figure 1). Blood tests indicated the following results: the white blood cell count was 7.9 × 10^9^/L, the percentage of neutrophils was 81.2%, the T cell count was 414.51/μL, the B cell count was 34.21/μL, the NK cell count was 35.55/μL, the procalcitonin level was 0.17 ng/mL, the erythrocyte sedimentation rate was 21 mm, and the C-reactive protein count was 20.83 mg/L. Tests for HIV, syphilis, and hepatitis B virus (HBV) were all negative. A muscle abscess was diagnosed and confirmed through magnetic resonance imaging (MRI) (Figure 1). The affected musculature included the right distal posterior femoral muscle group and adjacent intermuscular compartments. An enlarged lymph node in the right inguinal region was observed on local ultrasound (Figure 1). Histopathology of the skin from the right lower extremity showed lymphocytic infiltration (Figure 2). Additionally, histopathology of the muscles and lymph node (Figure 2) showed inflammatory changes and reactive lymphoid hyperplasia.

Venous blood, skin biopsy specimen, muscle tissue, and right inguinal lymph node puncture tissue cultures revealed no bacterial or fungal growth. The incubation period for microbiological culture of tissue samples is 2 weeks for fungi and 3 days for bacteria. Venous blood cultures incubated for 5 days reported no bacterial growth. Neither specialized culture media nor extended incubation periods were employed. Due to limited experience with uncommon bacterial infections, potential diagnostic omissions may have occurred. mNGS of the venous blood and muscle samples revealed no microbes. Finally, on day 13 after admission, mNGS of lymph node puncture tissue revealed *N. asteroides*. Microbiological analysis identified *Nocardia* at the genus level with 19 detected sequences, representing a relative abundance of 0.5% within the sampled microbial community. At the species level, *Nocardia asteroides* was specifically confirmed through eight distinct sequences. Notably, this isolate exhibited an RPM-ratio of 12.56, indicating significantly elevated detection relative to background microbial abundance (where RPM-ratio >1 suggests biological relevance). No other potential pathogens were detected. The reason why our patient developed a *Nocardia* abscess in the thigh remains unclear. The patient recalled an episode of temporary swelling and tenderness in the right thigh following friction with the ground 6 years ago. It is possible that our patient was infected with *Nocardia* at that time and developed the abscess after 6 years because of a latent infection. Why this infection developed 6 years post-trauma is difficult to explain. Whole-exome sequencing of peripheral blood showed that the patient had a monoallelic mutation in the lipopolysaccharide-responsive beige-like anchor protein (LRBA) gene. Genetic analysis revealed a compound variant in LRBA consisting of two heterozygous missense mutations: p.Val2066Met (c.6196G > A) and p.Ser952Leu (c.2855C > T). The initial empiric antimicrobial regimen for our patient was oral trimethoprim-sulfamethoxazole (TMP-SMX) (960 mg twice daily). During the first 3 days following admission, intravenous immunoglobulin (IVIG) was administered daily at a dose of 25 grams.

**Figure 3:**
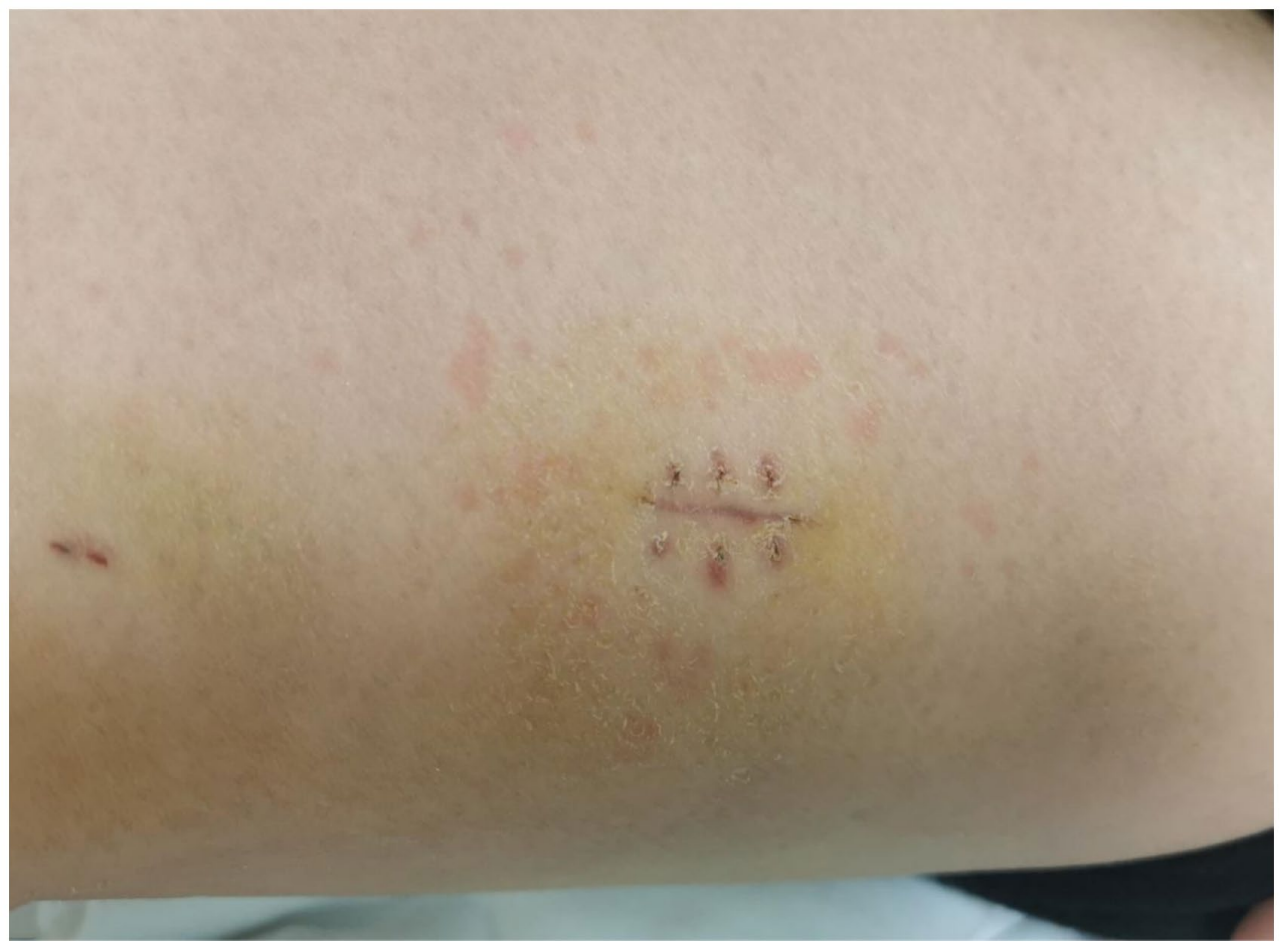
Erythema after 28 days of treatment.

During the first 4 days of admission, the patient was successively administered TMP-SMX, azithromycin, minocycline, and itraconazole capsules. No significant clinical improvement was observed. Consequently, all medications except TMP-SMX were discontinued. On day 5, treatment was initiated with intravenous voriconazole and moxifloxacin. This regimen was subsequently discontinued following the onset of transient xanthopsia and the development of generalized erythema in conjunction with high fever. Current management is restricted to maintaining TMP-SMX therapy. The patient’s body temperature returned to normal by the 9th day of hospitalization. In addition, the laboratory tests, MRI, and ultrasound were in remission. During the third week of hospitalization, IVIG was readministered at 25 grams daily for three consecutive days. By the 28th day of hospitalization, the area of erythema on the medial aspect of the right thigh had decreased in size (Figure 3). Her mental state was markedly improved. The patient continued to be treated with oral TMP-SMX (960 mg twice daily for 1 year). The lesion on the limb disappeared completely without recurrence.

## Discussion

Common species of *Nocardia* include *N. asteroides, N. brasiliensis*, *N. farcinica*, *N. otitidiscaviarum*, and others, all of which may cause various diseases in humans. Primary cutaneous nocardiosis is most commonly caused by *N. brasiliensis* (5). Skin involvement in nocardiosis can be classified into four categories: primary cutaneous, lymphocutaneous, cutaneous manifestations of disseminated *nocardia*, and mycetoma. Primary cutaneous infection most often results from direct inoculation of the bacteria due to trauma or surgery (6, 7). *N. asteroides* is one of the most common pathogenic species within the *Nocardia* genus, known for causing localized and systemic infections. Cutaneous nocardiosis typically presents as abscesses, cellulitis, or mycetoma, with *N. asteroides* being a frequent etiological agent. Cutaneous manifestations of *Nocardia* have been reported in immunocompetent hosts. However, involvement of the muscle layer is rare, with only three previously reported cases, due to *Nocardia brasiliensis* and *Nocardia farcinica* (8–10). Despite its clinical significance, diagnosis is often delayed due to its resemblance to other pyogenic or granulomatous infections. *Nocardia* infection should be suspected in cutaneous infections that respond poorly to routine treatment.

The traditional diagnostic method for nocardiosis involves culturing the bacteria. The microbiological laboratory should be informed, as *Nocardia* species grow poorly on many culture media and need long-term incubation periods (11). The conventional culture-based identification of *Nocardia* species typically requires 2–7 days of incubation, potentially delaying definitive diagnosis. This diagnostic challenge is further compounded by the fact that the majority of nocardiosis patients receive empirical antibiotic therapy prior to specimen collection, which significantly diminishes microbial culturability (12). mNGS is a novel approach characterized by high sensitivity, rapid detection, and reduced susceptibility to interference from prior antibiotic usage (13, 14). mNGS can theoretically detect all types of pathogens, which makes it suitable for difficult and atypical infectious diseases (14). mNGS demonstrates superior sensitivity for *Nocardia* detection compared to conventional methods, especially in culture-negative cases. While not yet replacing culture, it serves as a powerful complementary tool for rapid diagnosis. Ongoing improvements in sequencing depth and bioinformatics will likely increase detection rates further. However, mNGS also has some limitations. Its high cost can be a burden for some patients. Moreover, the time of sample collection may influence its sensitivity. A recent study revealed that 14 samples were identified to be *Nocardia* spp. positive by mNGS, whereas only five of them yielded positive culture results (15). This patient’s negative blood culture may be due to the influence of prior antibiotic usage.

Given the *Nocardia* infection, the patient should be evaluated for underlying primary immunodeficiency (PID) or inborn immunity errors (16). LRBA deficiency was described as a novel primary immunodeficiency (PID) in 2012 (17). Patients with LRBA deficiency present with a broad spectrum of clinical phenotypes, including autoimmunity, enteropathy, hypogammaglobulinemia, and recurrent infections (18). Bi-allelic mutations in the *LRBA* gene represent an important but underrecognized risk factor for nocardiosis. Previous studies have documented the identification of patients harboring heterozygous LRBA mutations in patient cohorts. These individuals were observed to exhibit significantly milder clinical manifestations compared to those with biallelic mutations. Their phenotype aligns with milder forms of common variable immunodeficiency (CVID) or CVID-like presentations, characterized by features such as selective antibody deficiency, later disease onset, predominant yet milder autoimmune manifestations, and the absence of severe complications (19–21). Increased awareness, improved diagnostic tools, and personalized management approaches are needed for this patient population.

Treatment of nocardiosis is tailored to the individual patient (22). Currently, TMP-SMX is commonly used as the primary medication for treating *Nocardia* (23). In some cases, such as mycetoma and deep abscesses, a longer duration of therapy, potentially up to a year, may be necessary (2, 24). The duration of treatment should be based on the patient’s immune status and the extent of the disease. Early recognition and effective therapy are imperative for achieving successful outcomes.

## Conclusion

In conclusion, *Nocardia* should be included in the differential diagnosis of cutaneous infections, particularly when there is no improvement with traditional antimicrobial regimens and when the infection is spreading into deeper muscle tissues. Both immunocompromised and immunocompetent patients are at risk. mNGS could help identify clinical infectious disease pathogens, especially in cases of culture-negative infections.

## Data Availability

The raw data supporting the conclusions of this article will be made available by the authors, without undue reservation.
